# Lung ultrasonography of pulmonary complications in preterm infants with respiratory distress syndrome

**DOI:** 10.3109/03009734.2011.643510

**Published:** 2012-02-15

**Authors:** Jovan Lovrenski

**Affiliations:** Radiology Department, Institute for Children and Adolescents Health Care of Vojvodina, Novi Sad, Serbia

**Keywords:** Complications, infant, lung, respiratory distress syndrome, ultrasonography

## Abstract

**Aim.:**

To evaluate the diagnostic possibilities of lung ultrasonography (LUS) in detecting pulmonary complications in preterm infants with respiratory distress syndrome (RDS).

**Material and methods.:**

A prospective study included 120 preterm infants with clinical and radiographic signs of RDS. LUS was performed using both a transthoracic and a transabdominal approach within the first 24 h of life, and, after that, follow-up LUS examinations were performed. In 47 detected pulmonary complications of RDS (hemorrhage, pneumothorax, pneumonia, atelectasis, bronchopulmonary dysplasia), comparisons between LUS and chest X-ray (CXR) were made. Also, 90 subpleural consolidations registered during LUS examinations were analysed. Statistical analysis included MANOVA and discriminant analysis, *t*-test, confidence interval, and positive predictive value.

**Results.:**

In 45 of 47 instances the same diagnosis of complication was detected with LUS as with CXR, indicating a high reliability of the method in premature infants with RDS. The only two false negative findings concerned partial pneumothorax. The positive predictive value of LUS was 100%. A statistically significant difference of LUS findings between the anterior and posterior lung areas was observed in both right and left hemithoraces.

**Conclusions.:**

LUS enables the detection of pulmonary complications in preterm infants with RDS and has the potential to reduce the number of CXRs. The specific guidelines for its use should be provided in a more extensive study.

## Introduction

Reduction of the dose of ionizing radiation (IR) is one of the main goals of contemporary paediatric radiology. Thus, the continuous search for the balance between the potential benefits and the potential delayed adverse effects, which may arise from the use of diagnostic procedures based on IR, is inevitable when working with children. This should certainly be one of the main topics of mutual co-operation between paediatricians, paediatric surgeons, and paediatric radiologists.

The risk of the effects of IR is higher the younger the child is (1,2); with the same dose of ionizing radiation, a 1-year-old child is 10–15 times more at risk of developing carcinoma than an adult (3). Nevertheless, there is a trend in children’s hospitals of increasing the number of radiographs and CT examinations by about 25% (4).

Of the many complications of prematurity (intracranial hemorrhage, necrotizing enterocolitis, sepsis, and retinopathy of prematurity), lung diseases, such as respiratory distress syndrome (RDS) and its complications (pulmonary hemorrhage, pneumonia, atelectasis, pneumothorax, air leak syndrome, and bronchopulmonary dysplasia (BPD)), remain the most common cause of neonatal morbidity. RDS is the clinical expression of surfactant deficiency in neonates and is typically presented with tachypnea, expiratory grunting, nasal flaring, cyanosis, and substernal and intercostal retractions (5).

The aim of this study was to evaluate the diagnostic possibilities of lung ultrasonography (LUS) in detecting pulmonary complications in preterm infants with RDS and in reducing the number of chest X-rays (CXRs).

### Normal LUS findings

When using the transthoracic approach, the pleura is visualized as a smooth, echogenic line. Since ultrasonography (US) is a dynamic examination, the evaluation of the pleura has to include the ‘lung sliding’ sign, which represents the sliding of the visceral pleura over the parietal pleura (6). Its absence is the main US criterion of pneumothorax (7).

**Figure 1.**
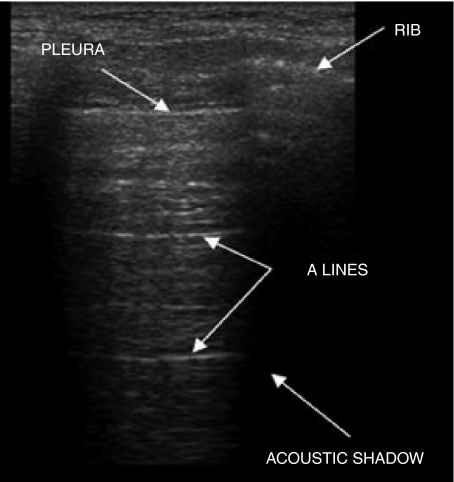
Normal lung US finding in a longitudinal section.

Beneath the pleura the lungs are filled with air, which disables visualization of the lung parenchyma. However, the high acoustic impedance between the visceral pleura and the lung parenchyma results in horizontal artefacts, which are the parallel echogenic lines below the pleural line, equally distanced from one another, and are called A lines (Figure 1) (8,9).

If the US examination of the lung bases is performed transabdominally, this examination is normally based on the acoustic phenomenon of ‘mirror image’, that is supradiaphragmatic projection of the liver or spleen (10,11).

### Pathological LUS findings

**Figure 2.**
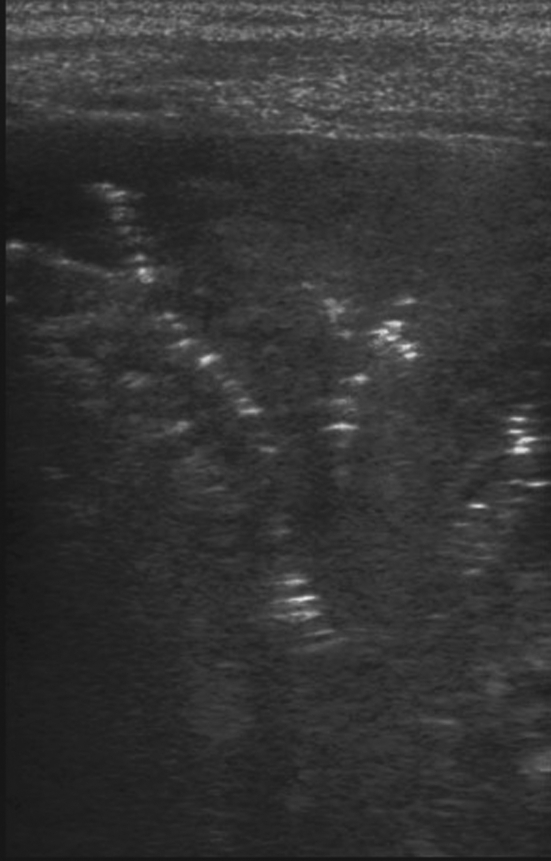
Dichotomous branching of the peripheral bronchioles within the subpleural consolidation.

**Figure 3.**
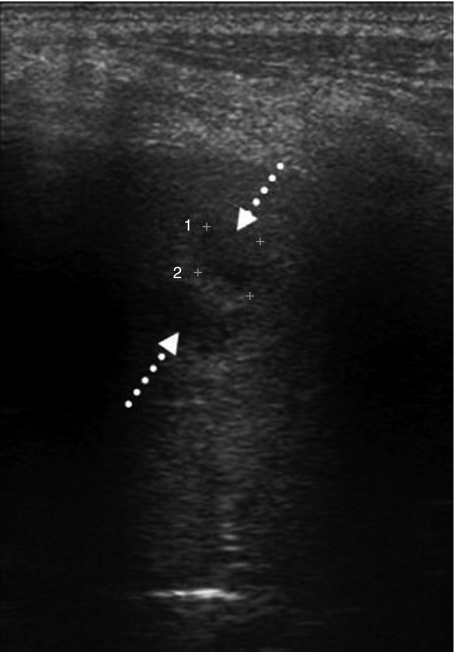
Two microabscesses (dotted arrows) with the greatest diameter of 7 mm.

**Figure 4.**
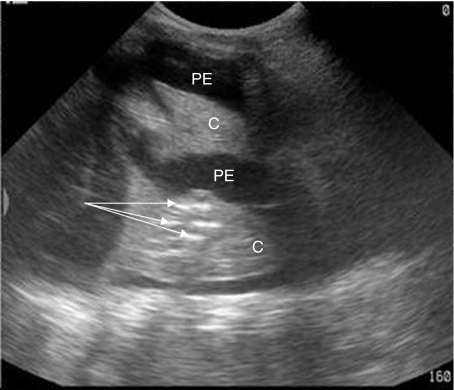
Parallel air bronchogram (arrows) within the pulmonary consolidation (C) of passive atelectasis due to large amount of pleural effusion (PE).

When the parenchymal disease propagates to the pleura, an acoustic window is formed using either a transthoracic or a transabdominal approach. This enables transmission of an ultrasound beam and evaluation of lung tissue. The absence of alveolar air in the lung periphery is visualized in the form of subpleural consolidation. Within the consolidation multiple branching of echogenic linear structures can be seen, which corresponds to the air bronchogram (Figure 2). The advantage of US examination is that it allows for visualization of the microabscess (Figure 3). Unlike in pneumonia, where the air bronchogram is branched, with passive atelectasis, mostly due to pleural effusion, it may become ‘compressed’ and parallel before the air has been fully reabsorbed (Figure 4) (12).

**Figure 5.**
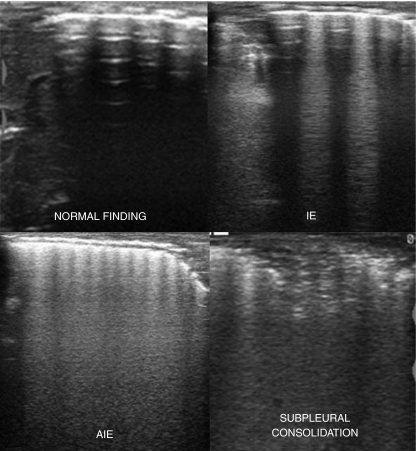
Four basic patterns of the lung US findings (IE = interstitial edema; AIE = alveolar-interstitial edema).

Also, a pathological finding is presented with vertically oriented ‘comet-tail’ artefacts in the lungs, which extend from the pleural line to the bottom of the screen. They are hyperechogenic, clearly defined, erase the A lines, move with ‘lung sliding’, and are called the B lines. They are a result of the accumulation of fluid in the subpleural interlobular septa surrounded by air (13,14). Their presence excludes pneumothorax as a diagnosis (8). Depending on the amount and distance of B lines, interstitial edema (IE) and alveolar-interstitial edema (AIE) can be recognized (Figure 5) (13).

Currently, LUS is not widespread in the detection of neonatal respiratory diseases. There have been just a few studies dealing with the diagnostic possibilities of US in this field (11,15–19). To our knowledge, a study searching for possibilities of US the detection of pulmonary complications (except BPD (15,17)) in preterm infants with RDS has not been published yet.

## Materials and methods

A prospective study was carried out at the Institute for Children and Adolescents Health Care of Vojvodina (ICAHCV) in Novi Sad, Serbia, in association with the neonatal intensive care unit (NICU) and the radiology department.

The inclusion criteria were clinical and radiographic signs of RDS, and gestational age (GA) under 37 weeks. The study included 120 preterm infants (weeks of gestation (WG) ranging from 23.86 to 36.89, mean value: 30.97 WG (SD 3.16, confidence interval (CI) 30.4–31.55 WG); birth weight, mean value: 1594.9 g (SD 626.79, CI 1481.59–1708.23 g)). The average age of the infants at the moment of admission to the ICAHCV was 16.29 h (CI 11.59–21 h).

Out of 120 premature infants, 51 (42.5%) received corticosteroids prenatally. Sixty-seven (55.83%) infants were born vaginally, while 53 (44.17%) were delivered by C-section. Surfactant was endotracheally administered in 85 (70.83%) patients (1 dose (49 patients), 2 (27 patients), 3 (8 patients), and 4 (1 patient)), with the first dose given at the average time of 3.74 h (CI 2.35–5.12 h). Mechanical ventilation was required in 95 patients, with mean duration of mechanical ventilation of 9.4 days (CI 5.49–13.31 days).

The Ethical Committee of the ICAHCV approved the research (date of issue: 1 February 2008, registration number: 185/7), and informed consent was obtained from the parents of each examined preterm infant.

**Figure 6.**
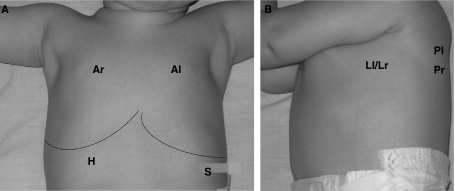
Examined lung areas in supine (A) and lateral decubitus (B) positions (H = transhepatic approach; S = transsplenic approach; Ar = anterior right, Al = anterior left, Lr = lateral right, Ll = lateral left, Pr = posterior right, and Pl = posterior left lung areas).

LUS examinations were performed by the same experienced paediatric radiologist (J.L.), using a 7.5 MHz linear probe (Sonoline Adara, Siemens, Erlangen, Germany) and both transthoracic and transabdominal approaches. The transthoracic US approach included examination in supine and both lateral decubitus positions of the anterior (between the sternum and the anterior axillary line), lateral (between the anterior and posterior axillary lines), and posterior (between the posterior axillary line and the spine) lung areas in caudo-cranial direction. The transabdominal US included the transhepatic and transsplenic approach in supine position to examine both lung bases. This US technique provided division of each hemithorax into four lung areas, i.e. eight lung areas per patient. The right lung base was examined by transhepatic approach (H), while right anterior (Ar), lateral (Lr), and posterior (Pr) lung areas were examined by transthoracic approach. The left lung base was examined by transsplenic approach (S), and transthoracic approach was used to examine left anterior (Al), lateral (Ll), and posterior (Pl) lung areas (Figure 6). Longitudinal, transversal (intercostal), and oblique sections were used.

LUS was performed within the first 24 h of life in all preterm infants included in the study. The double lung point sign, characteristic of transient tachypnea of the newborn (TTN), was excluded in each patient as a possible cause of respiratory distress (19). After that, follow-up examinations were performed until completely normal neonatal lung scans were obtained, until discharge from the hospital, or until the eventual fatal outcome. The approximate time of LUS examinations was 3–4 minutes, very often even less, and US gel was kept warm before the examinations, all in favour of keeping the thermal loss to a minimum.

The total number of US examinations was 512 (mean value 4.61), while 612 chest X-rays (CXR) (mean value 5.1) were clinically indicated during the research. During 8 out of 512 LUS examinations performed the oxygen saturation level dropped to 80%, which was an indication for immediate termination of the examination, whereby all infants stabilized instantly in saturation. At the time of examination all these eight patients were on mechanical ventilation.

The largest number of CXRs was found in the group of patients with BPD, with the mean value of 10.3 (SD 8.54). BPD was defined as a supplemental oxygen requirement beyond 28 days of age, with severity of the disease being based on the supplemental oxygen requirement in relation to 36 weeks’ postconceptional age (WPCA) (20). Radiographic signs of BPD varied from bilateral, ill-defined pulmonary opacities predominantly perihilar and homogeneous, to coarse reticulation characterized by streaky densities interspersed with small cystic lucencies.

In 47 pulmonary complications of RDS (hemorrhage, pneumonia, atelectasis, pneumothorax, and BPD), it was possible to compare the LUS findings with the CXRs, based on the criteria concerned with the time interval between them (CI 3.24–4.96 h), as well as with absence of all the clinical and therapy procedures that could influence and change the pulmonary findings. CXRs were interpreted by the same paediatric radiologist who performed LUS examinations, and who was blinded to the CXR and clinical findings before finishing the US report, in order to increase the objectivity of the study.

Ninety subpleural consolidations registered during LUS examinations were distributed in relation to their clinical and radiographically determined origin, and their size was statistically analysed.

The analysis included also every LUS examination with regard to the comparison of findings between anterior and posterior lung areas.

Statistical analysis included mean value, standard deviation (SD), confidence interval (CI), positive predictive value, multivariate analysis (MANOVA and discriminant function analysis), and univariate analysis (ANOVA *t* test, Roy’s *t*-test, Pearson’s contingency coefficient (χ), and the multiple correlation coefficient (*R*)). Assessment of the statistical significance was done using the *P* Value. A *P*-value less than 0.05 was considered statistically significant.

## Results

Out of 47 comparisons between LUS and CXR findings in our study, there have been 2 false negative findings in detecting pulmonary complications of RDS. Those two findings both concerned partial pneumothorax. The rest of 45 comparisons showed a complete match between CXR and LUS findings. Forty-three of them were positive in both diagnostic modalities, and two findings were normal, i.e. they indicated a complete lung re-expansion of the previously radiographically confirmed pneumothorax.

**Figure 7.**
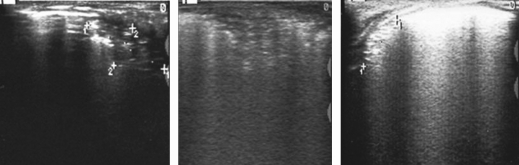
Inability of the US to differentiate between subpleural consolidations stemming from: pneumonia (left image), atelectasis (middle image), and hemorrhage (right image).

The positive predictive value of the US method in the diagnostics of pulmonary complications in premature infants with RDS was 100%. However, ultrasonographic verification of the pathological findings in the premature’s lungs could not give a differentiation between the origins of the subpleural consolidations based on its US features (Figure 7).

The origin of 90 US registered subpleural consolidations was as follows: RDS (*n* = 37 consolidations), pulmonary hemorrhage (*n* = 11), pneumothorax (*n* = 3), pneumonia (*n* = 20), atelectasis (*n* = 8) and BPD (*n* = 11).

Among 90 subpleural consolidations ultrasonographically detected in the study, the highest average sizes (av.) were found with the consolidations stemming from: BPD (av. 27.41 mm, SD 8.23 mm, CI 21.88–32.94 mm), pneumonia (av. 26.95 mm, SD 8.40 mm, CI 23.02–30.88 mm), and atelectasis (av. 24.50 mm, SD 6.37 mm, CI 19.17–29.83 mm). Lower average sizes were found with the consolidations stemming from pulmonary hemorrhage (av. 19.86 mm, SD 9.77 mm, CI 13.30–26.43 mm) and RDS (av. 21.08 mm, SD 9.23 mm, CI 18–24.16 mm).

MANOVA and discriminant analysis showed a statistically significant difference (*P* < 0.05) between LUS findings of the anterior and posterior lung areas, in both right and left hemithoraces (Ar versus Pr; Al versus Pl). A comparison of LUS findings showed a normal finding dominating anterior lung areas, while both compact B lines presenting AIE and subpleural consolidations were represented more within the posterior lung areas (Table I).

**Table I.** Distribution of different LUS findings in anterior and posterior lung areas of both hemithoraces, with evaluation of statistical significance.

US finding	Lung areas	%	*P* Value
Normal	Ar versus Pr	24.7 versus 5.5	< 0.05
Normal	Al versus Pl	20.5 versus 6.8	< 0.05
AIE	Ar versus Pr	27.4 versus 34.2	NS
AIE	Al versus Pl	23.3 versus 38.4	NS
SC	Ar versus Pr	1.4 versus 17.8	< 0.05
SC	Al versus Pl	4.1 versus 16.4	< 0.05

AIE = alveolar-interstitial edema; SC = subpleural consolidation; Ar = anterior right lung area; Al = anterior left lung area; Pr = posterior right lung area; Pl = posterior left lung area; NS = non-significant.

## Discussion

The continuous balancing between the potential harmful effects of diagnostic procedures based on X-rays and their potential benefits is especially highlighted in paediatric radiology.

Although the cumulative effects of relatively low doses of IR are not fully understood, there are studies that suggest up to three times higher probability of occurrence of acute lymphoblastic leukaemia in children with three or more diagnostic procedures based on X-rays, among which plain radiographs were the most common ones, and not CT or fluoroscopy (21–23). These data place the population of premature infants in the group of high vulnerability and risk of developing late sequelae induced by IR, as these children have a very wide range of complications with high diagnostic requirements, so the exposure to X-rays in NICU is usually not their last one.

Given the possibility of evaluating pleural lesions, lungs, and extracardiac mediastinum, LUS in children has been recognized as a potentially useful diagnostic method (6,24–31). This is supported by the fact that children have a thinner thoracic wall, and smaller width of the thorax and lung volume, which enables a better image quality and visualization of almost the entire surface of the lungs compared to the adult population (19). Also, the relative simplicity of mastering the basic LUS patterns is of great importance. Bedetti et al. have demonstrated this by showing how inexperienced doctors can learn to detect IE after ten examinations, i.e. after only 30 minutes of training, so LUS is not considered to be an examination of high inter- and intraobserver variability in the interpretation (18,32). There is, on the other hand, often a significant variation in inter- and intraobserver interpretation of the same CXR images, which very much depends on the radiologist’s experience (33). Nevertheless, experience may play an important role in LUS examinations. An experienced paediatric radiologist can perform this examination faster, which is extremely important in such a vulnerable population as preterm infants are. This is probably the main reason for only eight complications in our 2-year study. Each patient’s condition was stabilized promptly after terminating the LUS examination.

In our study the diagnostics of pulmonary complications of RDS showed a positive predictive value of 100%, which indicates the reliability of positive LUS findings. Also, according to our everyday experience, LUS has often provided a more accurate detection and localization of the pathological changes in the lungs compared with CXRs and has proved to be very reliable in monitoring the clinical changes. It has also been demonstrated in adults with AIE that LUS has a higher sensitivity than CXR (34).

Although the US diagnostic features of pneumothorax are well known (7,8,35,36), our research has shown that partial pneumothorax might be a diagnostic problem, and so when suspecting the development of pneumothorax we would advise a CXR. However, what is extremely important is the ability of ultrasonography easily to exclude pneumothorax as a possible diagnosis or to register a complete re-expansion of the lungs, based on the presence of the ‘lung sliding’ sign, as well as B lines (7,8). Given the above, our suggestion is that the results of the pneumothorax drainage should be verified by ultrasound first, avoiding CXR examinations whenever it is possible.

The largest size of subpleural consolidations was registered in patients with BPD, which due to the extensity of pathological changes was to be expected (5). They were mostly having coarse reticulation and alveolar consolidations dominating the CXRs. The lowest values in consolidation size registered in pulmonary hemorrhage were also expected, considering that the hemorrhages are not always extensive and are often manifested with only small alveolar consolidations (5).

Although there are some possibilities of differentiation between the origins of the subpleural consolidations based on its US features in the adult population (12,31,36,37), our research has not demonstrated this possibility in prematures. For example, the subpleural consolidations stemming from pneumonia, atelectasis, and hemorrhage did not show US differences which would provide a diagnosis. In our experience, it is sometimes difficult to distinguish these consolidations on CXRs too, especially when the paediatric radiologist does not have clinical information available. This indicates the necessity of daily co-operation and communication between clinicians and radiologists, in order to achieve the most adequate interpretation of radiological findings.

As we have noticed during the study that the pathological pattern is most often found and is the slowest to regress in the posterior lung areas, a comparison was made between the US findings of the anterior and posterior lung areas on both sides. Anterior lung areas on both sides showed a statistically significant occurrence of normal findings, while the pattern of compact B lines presenting AIE and subpleural consolidations were represented more in the posterior lung areas. These results might be a consequence of the patient’s often continuous supine position, which causes reduced ventilation in the posterior lung areas and the longest retention of interstitial fluid in these parts of the lung. The US findings may be of great significance in everyday practice, especially in the period after the extubation, as LUS could point out the poorly ventilated areas of the lung parenchyma.

Although the advantages of LUS are numerous (non-ionizing, can be performed and repeated at the bedside, low-cost technology, relatively simple to learn), we have to be aware of its limitations. There are some acute complications of RDS secondary to air leak syndrome (pneumomediastinum, interstitial emphysema, pneumopericardium) that cannot be detected using LUS (18).

In conclusion, LUS enables a detection of pulmonary complications in preterm infants with RDS. We believe that LUS in combination with clinical parameters has the potential to reduce the number of CXRs in NICUs.

### Limitations of the study

Even though blinded for the specific CXR and clinical findings prior to each LUS examination, both diagnostic modalities have been evaluated by the same investigator, which makes bias difficult to exclude entirely. The investigator was the only experienced paediatric radiologist in the field of LUS. Also, it was not possible to conduct a blinded, independent survey of these examinations in this very demanding and time-consuming study due to a small number of paediatric radiologists in our hospital, which is the only children’s hospital in this region.

Since US scans were performed by a single experienced operator, it is reasonable to hypothesize that similar results might not be immediately achieved by less experienced operators.

Therefore, on the basis of these limitations, it is necessary to conduct a more extensive, preferably multicentre study, which could confirm the findings of this study and provide specific guidelines for the use of LUS in premature infants.
